# Optimal timing of surgery for prenatally diagnosed choledochal cysts

**DOI:** 10.3389/fped.2023.1308667

**Published:** 2023-11-23

**Authors:** In Geol Ho, Kyong Ihn, Ho Jong Jeon, Dong Eun Lee, Seok Joo Han

**Affiliations:** ^1^ Division of Pediatric Surgery, Department of Surgery, Yonsei University College of Medicine, Severance Children’s Hospital, Seoul, Republic of Korea; ^2^ Division of Pediatric Surgery, Department of Surgery, National Health Insurance Service Ilsan Hospital, Goyang, Republic of Korea

**Keywords:** choledochal cyst, antenatal diagnosis, surgical treatment, optimal timing, prognosis

## Abstract

**Objective:**

Choledochal cysts are increasingly being diagnosed antenatally. The appropriate time of surgical treatment has the greatest impact on the prognosis of choledochal cyst treatment. The purpose of this study was to compare the clinical outcomes of prenatally diagnosed choledochal cysts in infants according to the surgical treatment timing.

**Methods:**

We retrospectively reviewed the medical records of infants who underwent surgery for choledochal cysts with antenatal diagnoses. We investigated each patient's demographic information, type of choledochal cyst, serum liver enzyme levels, and surgical outcomes according to the surgical intervention timing.

**Results:**

Between May 2006 and December 2020, 93 infants underwent surgery to treat choledochal cysts; among them, 68 had antenatally suspected choledochal cysts. Of the 68 patients, 21 developed symptoms directly after birth. While 38 patients remained asymptomatic, 9 developed symptoms before operation. To compare surgical outcomes, asymptomatic patients were divided into early (13 cases) and late (25 cases) operation groups based on an age benchmark of 30 days. The early surgical group experienced longer times to resume a full diet (6.0 ± 1.6 vs. 4.5 ± 0.7, *p* < 0.001) and longer postoperative hospital stays (11 ± 3.9 vs. 7.5 ± 0.8, *p* < 0.001). Surgical complications occurred in two patients in the early operation group. Minimally invasive surgery was performed in 12 patients in the late operation group. In both groups, postoperative liver function recovered at 6 months, with no significant difference.

**Conclusion:**

The results of this study showed longer hospital stays, increased diet durations, and postoperative complications in early surgery patients. However, liver function recovery was not different between the early and late operation groups. Thus, asymptomatic patients should be closely monitored, and we recommend that definitive surgical intervention be postponed until 4 months of age or until weight reaches 7 kg.

## 1. Introduction

Advances in prenatal ultrasonography have led to an increase in antenatally diagnosed congenital biliary dilatation, including choledochal cysts (CCs) (1, 2). Patients with prenatal diagnoses may experience rapid development of hepatic damage and symptoms due to protein plugs that form in the bile duct because of pancreatobiliary reflux and obstruction of the distal duct (3, 4). In patients with CCs who experience symptoms after birth, depending on their clinical conditions, early surgical intervention is essential. Conversely, the optimal timing of surgical intervention for those antenatally diagnosed with asymptomatic CCs remains open to debate (5–7). Suita et al. reported that most patients prenatally diagnosed with CCs were asymptomatic when referred to tertiary pediatric institutions (8), and few reports have formulated a management plan for antenatally diagnosed asymptomatic CCs before the onset of symptoms (9, 10). In the case of asymptomatic CCs, the postponement of surgical intervention until patients grow might be a valid option. However, the optimal timing of surgical intervention for asymptomatic antenatally diagnosed CCs is not clearly defined. This study aimed to identify the optimal surgical time by comparing the surgical outcomes of antenatally diagnosed asymptomatic CCs according to surgical treatment timing.

## 2. Patients and methods

### 2.1. Patients

**Figure 1:**
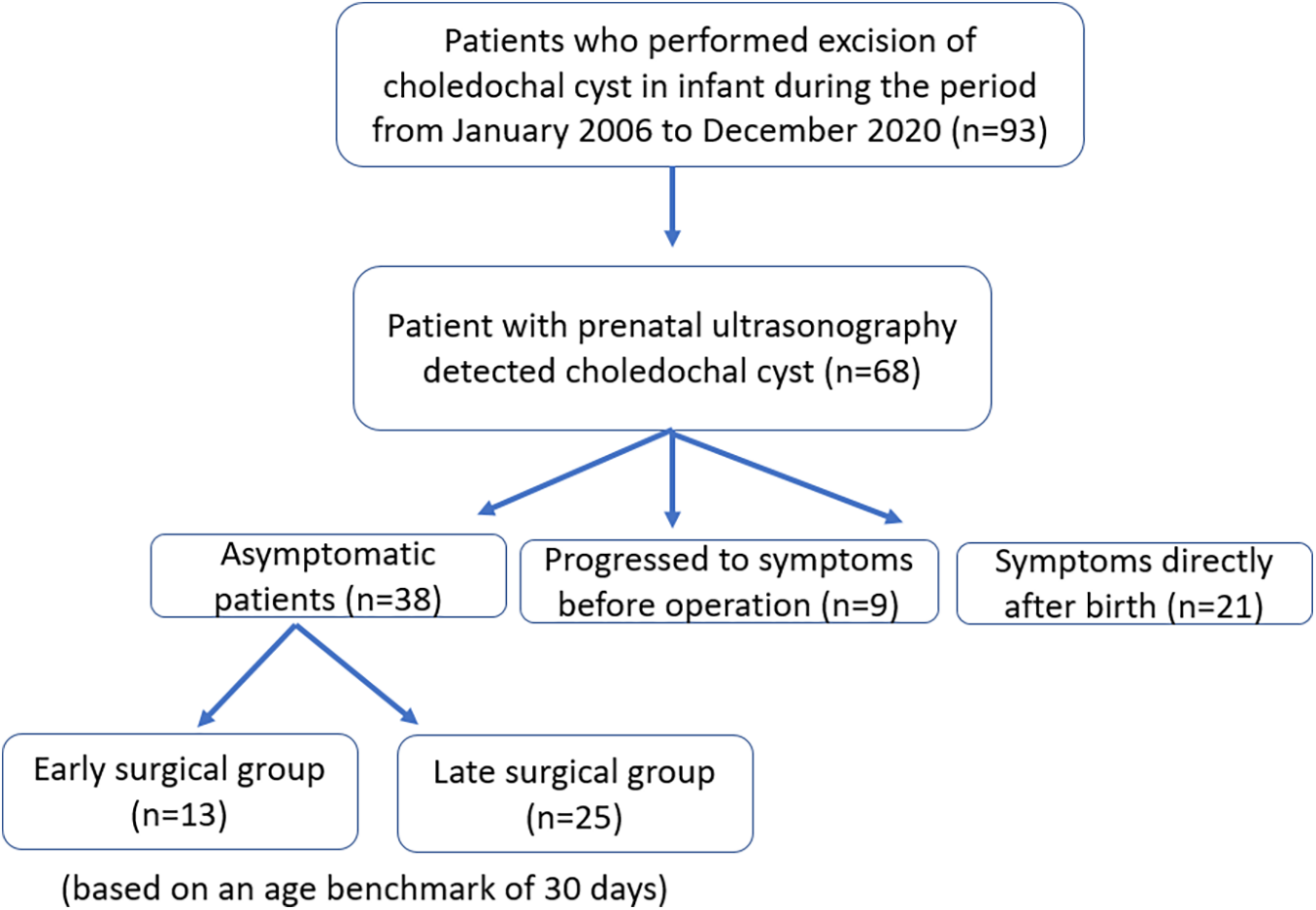
Flowchart of the study.

We retrospectively analyzed the medical records of patients who underwent surgical treatment for CCs with antenatal diagnoses at the Severance Children's Hospital. Between January 2006 and December 2020, 93 infant patients underwent surgical treatment of CCs, and among them, 68 patients were antenatally diagnosed with CCs (Figure 1). Among these 68 patients, 38 were asymptomatic, 9 developed symptoms, and 21 experienced symptoms directly after birth. First, asymptomatic patients were divided into early and late operation groups based on the benchmark age of 30 days. The surgical outcomes (time to return to a full diet, hospital stay, early and late surgical complications) were investigated. Second, we identified predictors of symptom development in asymptomatic CCs. This study was approved by the Institutional Review Board of the Yonsei University College of Medicine (4-2022-1616). The requirement for informed consent was waived because of the retrospective design.

### 2.2. Pre-operation evaluation and surgical indications

All patients underwent ultrasonography (US) and magnetic resonance cholangiopancreatography (MRCP) after birth to confirm the diagnoses of CCs and Todani types. Biochemistry laboratory tests were performed periodically to confirm obstructive jaundice. If patients had biliary obstruction and clinical symptoms including jaundice, acholic stool, vomiting, abdominal distension, irritable crying, and being difficult to console/comfort, they underwent surgery as soon as possible. Asymptomatic patients underwent an initial diagnosis exam and follow-up in an outpatient clinic. Regarding follow-up, a laboratory test was performed in the first and second weeks to check for obstructive jaundice, and in the fourth week, a laboratory test and US were performed to check for increases in cyst size and sludge. During the follow-up period, if clinical symptoms, obstructive jaundice, increase in cyst size, or sludge were observed, early surgical treatment was performed. Surgical treatment was not performed in the absence of the aforementioned observations, and patients were followed up until they reached an age of 4 months or a weight of 7 kg. We explained the surgical options (open or robotic) to the parents and proceeded with the surgery based on their decision.

### 2.3. Statistical analysis

All data were analyzed using IBM SPSS v.25 (IBM, Armonk, NY, USA). Continuous data are presented as means and standard deviations. Categorical data were compared using one-way ANOVA or the *t*-test for normally distributed data and the Mann–Whitney *U*-test for non-normally distributed data, and *p*-values < 0.05 were considered statistically significant.

## 3. Results

### 3.1. Demographics

**Figure 2:**
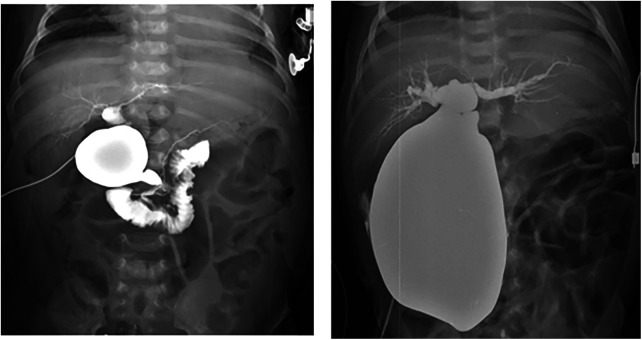
Operative cholangiography. (**A**) Non-obstructive cholangiopathy of the distal bile duct in an asymptomatic patient. (**B**) Obstructive cholangiopathy of the distal bile duct in a patient with symptoms directly after birth.

The demographic characteristics of patients who were antenatally diagnosed with CCs and underwent surgical treatment are summarized in Table 1. A total of 68 patients underwent treatment for CCs. Of the 68 patients, 38 (55.8%) remained asymptomatic, 9 (13.2%) developed symptoms before surgery, and 21 (30.8%) developed symptoms directly after birth. The patients had a median gestational age of 38 weeks (range, 31–41 weeks), and the mean birth weight was 3.6 ± 4.1 kg. The median prenatal diagnosis gestational age was 28 weeks (range, 15–38 weeks). A preoperative imaging evaluation using US and MRCP was performed in all patients. The mean cyst diameter (cm) in MRCP was 3.1 ± 1.3 cm, that of the asymptomatic group was 3.1 ± 1.3 cm, that of the group that developed symptoms before surgery was 3.4 ± 2.0 cm, and that of the group that developed symptoms directly after birth was 4.7 ± 2.0 cm. A significantly large cyst size was observed in the group that developed symptoms directly after birth (*p* = 0.004). The presence of anomalous pancreaticobiliary ductal union (APBDU) rate was 72% (49 cases), and no significant difference was observed between each group (28 vs. 8 vs. 13, *p* = 0.3). The Todani type classification was Ia in 22 cases (32.3%), Ib in 1 case (1.4%), Ic in 17 cases (25%), II in 2 cases (2.9%), III in 2 cases (2.9%), IV in 23 cases (33.8%), and V in 1 case (1.4%); no significant differences were observed between each group (*P = *0.14). An intraoperative cholangiogram was performed in 50 (73.5%) patients, and 29 (58%) patients had obstructive cholangiopathy of the distal bile duct. A significantly higher occurrence of obstructive cholangiopathy was observed in the group that developed symptoms before surgery and group that developed symptoms directly after birth (28% vs. 71.4% vs. 94.4%, *p* < 0.001. The mean follow-up period was 4.3 ± 2.6 years. Figure 2 shows the representative operative cholangiography in patients with non-obstructive cholangiopathy and with obstructive cholangiopathy.

**Table 1:** Characteristics of patient's antenatal diagnosis with choledochal cysts.

Characteristics	Total	Asymptomatic	Developed symptoms before surgery	Developed symptoms directly after birth	*p*-value
Cases (*n*)	68	38	9	21	
Sex (male: female)	21: 47	12: 26	2: 7	7: 14	0.93
Median IUP (weeks)	38 (31–41)	39 (34–41)	38 (31–40)	38 (31–41)	0.288
Mean body weight (kg)_birth	3.6 ± 4.1	3.1 ± 0.3	3.1 ± 0.9	3.1 ± 0.6	0.451
Prenatal diagnosis IUP (weeks)	28 (15–38)	28 (20–38)	27 (20–33)	27 (15–36)	0.871
Median age (days)_operation	35 (7–297)	87 (11–297)	58 (25–124)	13 (7–55)	<0.001
Mean body weight (kg)_operation	4.9 ± 2.0	5.9 ± 2.0	5.1 ± 1.2	3.2 ± 0.6	<0.001
Mean cyst diameters (cm)_MRCP	3.6 ± 1.5	3.1 ± 1.3	3.4 ± 2.0	4.7 ± 2.0	0.004
Presence of APBDU_MRCP (*N*, %)	49 (72%)	28 (73%)	8 (88%)	13 (61.9%)	0.302
Operative Cholangiography *(Obstructive cholangiopathy N, %)*	50 (29, 58%)	25 (7, 28%)	7 (5, 71.4%)	18 (17, 94.4%)	<0.001
Todani type					
I					
Ia	22	13	2	7	0.14
Ib	1	1	0	0	
Ic	17	12	1	4	
II	2	1	0	1	
III	2	1	1	0	
IV	23	10	4	9	
V	1	0	1	0	
Mean follow period (years)	4.3 ± 2.6	4.6 ± 2.7	4.9 ± 3.3	3.7 ± 1.9	0.342

IUP, Iintrauterine pregnancy; MRCP, magnetic resonance cholangiopancreatography; APBDU, anomalous pancreaticobiliary ductal union.

### 3.2. Clinical information and surgical outcomes of the asymptomatic group

**Figure 3:**
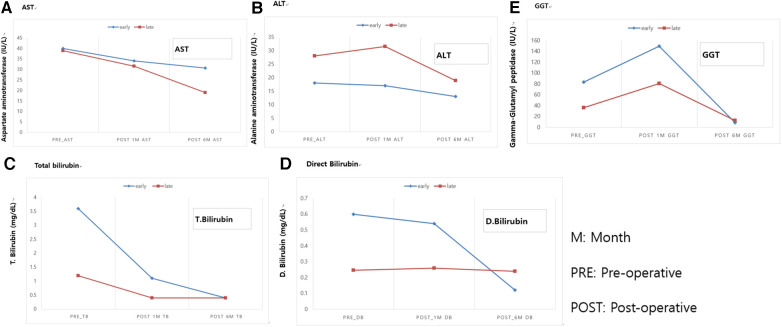
Comparison of postoperative blood liver enzyme recovery between asymptomatic patients with CCs who underwent early and late surgical interventions. (**A**) AST. (**B**) ALT. (**C**) Total bilirubin. (**D**) Direct Bilirubin. (**E**) GGT. M, month; PRE, pre-operative; POST, post-operative. AST, aspartate aminotransferase; ALT, alanine aminotransferase; GGT, Gamma glutamyl peptidase.

The clinical information for asymptomatic patients is summarized in Table 2. Patients were divided into early and late surgical groups based on the benchmark age of 30 days. In total, 13 of 38 patients were classified as the early surgical group (ESG) and 25 patients were classified as the late surgical group (LSG). The clinical characteristics of the ESG and LSG included the prenatal diagnosis time (24 vs. 28 weeks, *p* = 0.38), median gestational age (38 vs. 39 weeks, *p* = 0.137), presence of APDBU (9 vs. 19, *p* = 0.9), mean cyst diameter (3.5 ± 1.4 vs. 3.0 ± 1.2 cm, *p* = 0.205), and type of cyst (Todani type) (*p* = 0.09); no significant differences were observed between the groups. The LSG had a higher median age (13 vs. 135 days, *p* < 0.001) and mean body weight at operation (3.5 ± 0.4 vs. 7.1 ± 1.4 kg, *p* < 0.001) compared with the ESG. Table 3 describes the surgical outcomes of asymptomatic patients. All ESG patients underwent surgery with the open method, whereas in the LSG, 13 patients underwent surgery with the open method, and 12 patients underwent minimally invasive surgery (MIS, robotic method). The operation time was longer in the ESG than in the LSG (350.3 ± 56.6 vs. 296.3 ± 55.3, respectively, *p* < 0.022). Furthermore, the ESG had a longer time to resume a full diet (6.0 ± 1.6 vs. 4.5 ± 0.7 days, *p* < 0.001), drain duration (6.5 ± 1.6 vs. 5.1 ± 1.2 days, *p* = 0.009), and postoperative hospital stay (11 ± 3.9 vs. 7.5 ± 0.8 days, *p* < 0.001) compared with the LSG. No significant differences were observed in postoperative complications; however, complications occurred in two patients in the ESG. One patient had bile leakage, and one patient had a cavernous transformation of the portal vein. Serum liver enzyme levels improved significantly in both groups after the operation (Figure 3). Aspartate aminotransferase (AST), alanine aminotransferase (ALT), total bilirubin, direct bilirubin, and gamma glutamyl peptidase (GGT) were at normal levels at 6 months of follow-up. No difference was observed in the recovery of liver function according to the operation time.

**Table 2:** Clinical information of asymptomatic patients classified into early and late surgical groups.

Asymptomatic (*n* = 38)	Early surgical group (*n* = 13)	Late surgical group (*n* = 25)	*p*-value
Sex (male: female)	5: 8	7: 18	0.51
Median prenatal diagnosis IUP (weeks)	24 (20–31)	28 (20–38)	0.38
Median IUP (weeks)	38.5 (37–41)	39 (34–41)	0.137
Median age (days)_Operation	13 (11–26)	135 (34–297)	<0.001
Mean body weight (kg)_Operation	3.5 ± 0.4	7.1 ± 1.4	<0.001
Presence of APBDU	9 (70%)	19 (76%)	0.9
Mean cyst diameters (cm)_MRCP	3.5 ± 1.4	3.0 ± 1.2	0.205
Todani type			
I			
Ia	7	6	0.09
Ib	0	1	
Ic	3	9	
II	0	1	
III	0	1	
IV	3	7	
Preoperative serum bio-chemistry			
AST (IU/L)	40 ± 33.1	39.7 ± 18.7	0.18
ALT (IU/L)	18.6 ± 14.6	28.2 ± 21.4	0.98
Total bilirubin (mg/dl)	3.62 ± 2.6	0.6 ± 0.76	<0.001
Direct bilirubin (mg/dl)	0.62 ± 0.2	0.18 ± 0.14	0.15
GGT (IU/L)	83.5 ± 55.6	35.4 ± 27.9	0.004

IUP, intrauterine pregnancy; MRCP, magnetic resonance cholangiopancreatography; APBDU, anomalous pancreaticobiliary ductal union; AST, aspartate aminotransferase; ALT, alanine aminotransferase; GGT, gamma-glutamyl peptidase.

**Table 3:** Surgical outcomes of asymptomatic patients classified into early and late surgical groups.

Asymptomatic (*n* = 38)	Early surgical group (*n* = 13)	Late surgical group (*n *= 25)	*p*-value
Operation methods			
Open	13	13	0.012
Robotic	0	12	
Total operation time			
Open	350.3 ± 56.6	296.3 ± 55.3	0.022
Robotic	0	405.8 ± 64.2	
Time to resume full diet (days)	6.0 ± 1.6	4.5 ± 0.7	<0.001
Drain duration (days)	6.5 ± 1.6	5.1 ± 1.2	0.009
Postoperative hospital stays (days)	11 ± 3.9	7.5 ± 0.8	<0.001
Postoperative complication	2	0	
Early complication	1^a^	0	0.136
Late complication	1^b^	0	

^a^ Bile leakage.

^b^ Portal vein cavernous transformation.

### 3.3. Investigating the progression to symptomatic CCs

In our study, 9 (13.2%) of 68 patients developed symptoms before surgery. We identified indicators of symptom development through biochemical investigations and anatomical features of patients with symptoms at the time of diagnosis (Table 4). Individual serum liver enzymes of patients who remained asymptomatic and those of patients who developed CC-related symptoms were measured. Patients who developed symptoms before surgery had higher levels of AST (40.5 ± 24.2 vs. 119.6 ± 198.4, *p* = 0.019), ALT (25.3 ± 19.8 vs. 60.11 ± 70.8, *p* = 0.011), GGT (52.5 ± 45.6 vs. 318 ± 244.8, *p* < 0.001), total bilirubin (1.6 ± 2.2 vs. 4.6 ± 4.3, *p* = 0.005), and direct bilirubin (0.4 ± 0.2 vs. 1.6 ± 1.9, *p* < 0.001) compared with patients in the asymptomatic group. Anatomical features, such as the mean cyst diameter (3.2 ± 1.2 cm vs. 3.4 ± 2.0 cm, *p* = 0.638), presence of APBDU (27 vs. 10, *p* = 0.315), and Todani type (*p* = 0.134) were not significantly different between the groups. However, operative cholangiography was more obstructive in patients who developed symptoms before surgery (28% vs. 71.4%, respectively, *p* = 0.036).

**Table 4:** Indicators of biochemical and anatomical variants in asymptomatic patients and those who developed symptoms at the time of diagnosis.

	Asymptomatic	Progressed to symptomatic	*p*-value
AST (IU/L)	40.5 ± 24.2	119.6 ± 198.4	0.019
ALT (IU/L)	25.3 ± 19.8	60.11 ± 70.8	0.011
GGT (IU/L)	52.5 ± 45.6	318.2 ± 244.8	<0.001
Total bilirubin (mg/dl)	1.6 ± 2.2	4.6 ± 4.3	0.005
Direct bilirubin (mg/dl)	0.4 ± 0.2	1.6 ± 1.9	<0.001
Serum amylase (U/L)	14.3 ± 11.1	12.11 ± 11.3	0.602
Serum lipase (U/L)	20.3 ± 8.5	15.2 ± 8.4	0.115
Mean cyst diameters (cm)_MRCP	3.2 ± 1.2	3.4 ± 2.0	0.638
Present of APBDU_MRCP	27	10	0.315
Operative Cholangiography *(Obstructive cholangiopathy n, %)*	25 (7, 28.0%)	7 (5, 71.4%)	0.036
Todani type			
I			
Ia	13	2	0.134
Ib	1	0	
Ic	12	1	
II	1	0	
III	1	1	
IV	10	4	
V	0	1	

AST, aspartate aminotransferase; ALT, alanine aminotransferase; GGT, gamma glutamyl peptidase; MRCP, magnetic resonance cholangiopancreatography; APBDU, anomalous pancreaticobiliary ductal union.

## 4. Discussion

In this study, we evaluated the surgical outcomes of prenatally diagnosed CCs based on surgical timing. We found that among asymptomatic patients with CCs, the ESG had longer hospital stays, longer time to return to a full diet, and more postoperative complications than the LSG. However, serum liver enzyme recovery was not different between the ESG and LSG. Furthermore, biochemical test levels (AST, ALT, total bilirubin, direct bilirubin, and GGT) were significantly higher in patients who developed symptoms, and obstructive cholangiopathy was more common in patients who developed symptoms than in patients in the asymptomatic group.

The appropriate timing of surgical treatment for asymptomatic patients remains a dilemma for surgeons (2, 11–15). Missing the appropriate surgical time can lead to serious complications, such as ascending cholangitis, cyst rupture, and obstruction of the stomach outlet (16). Furthermore, a prolonged period of cholestasis in infants may lead to liver cirrhosis and portal hypertension (10). Based on the prognosis of CCs in children, the study of prenatal diagnosis of CCs and understanding of its pathophysiology are becoming of greater importance. Previous studies have emphasized that newborns and infants with CCs make up a limited population (7, 17). The etiology of CCs remains unknown; however, CCs are generally considered congenital (18). Chen et al. (19) reported that, compared with older patients with CCs, infants with CCs presented with different symptoms (painless jaundice), anatomical features (ending in a blind pouch of CCs), and normal levels of cystic amylase and lipase. The presence of blind-ending cysts or obstructive cholangiopathy of the distal bile duct, low bile amylase, and physiological deficiency of pancreatic exocrine function during the perinatal period suggests an alternate hypothesis for infantile CCs (7, 18).

**Figure 4:**
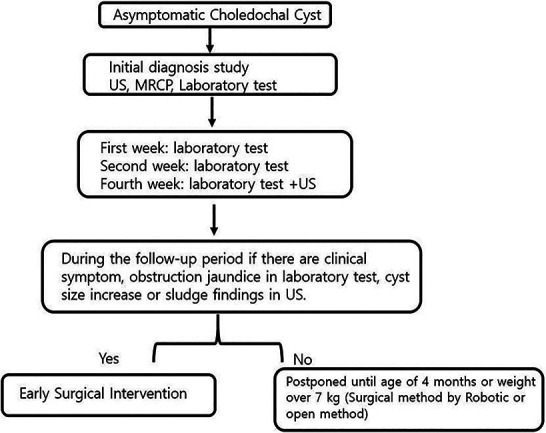
Follow-up flowchart for asymptomatic patients with choledochal cysts.

In our study, 21 (30.8%) patients developed symptoms directly after birth and 9 (13.2%) developed symptoms before surgical intervention. Our results suggest that the size of CCs was larger in symptomatic patients than in asymptomatic patients, and obstructive cholangiopathy was more common in patients who developed symptoms directly after birth and in those who developed symptoms before surgery; these results are consistent with the findings and assertions of previous studies (19, 20). Typically, obstructive jaundice and increased cyst size are indications for surgical intervention (21). In contrast, in asymptomatic patients with CCs, postponing elective definitive surgery is recommended to ensure safe anesthesia (2, 11, 12). Moreover, surgical intervention for the construction of a biliodigestive anastomosis in a small infant can be technically demanding. This issue may lead to a higher postoperative complication rate (15, 22, 23). Similarly, in our study, longer operation time and more surgical complications were observed in the ESG than in the LSG. In the Netherlands, researchers argued that surgery can be safely delayed until the age of 6 months or a weight of 6 kg (9). In a prospective, randomized study in China, researchers claimed that definitive surgery is feasible and safe for asymptomatic patients with CCs in the neonatal period (10). We agree with the need for early surgical intervention claimed in previous studies. However, determining the appropriate timing of surgical intervention while following up for a certain period after birth is important to reduce surgical complications and consider a long-term prognosis. In our study, the median age at surgery in the asymptomatic patient group was 87 days. Compared with that of other studies, the surgical operation time in our study was similar or slightly earlier. Our results also indicated that the LSG (median age ≥4 months and weight ≥7 kg) had shorter hospital stays, time to return to a full diet, and drain duration, as well as a lower incidence of surgical-related complications, than the ESG. However, in our study, MIS is included in the LSG. This is expected to introduce bias in the interpretation of surgical outcomes. Nevertheless, we also consider this as an advantage of late surgery. In our study, the robotic surgery system was performed as an MIS technique, which has the advantage of overcoming the limitations of conventional laparoscopic surgery (24, 25). In our study, the decision on LSG timing was influenced by MIS. In our experience, robotic surgery for pediatric choledochal cysts was performed safely when the patient's weight exceeded a minimum weight of 7 kg (25, 26). Accordingly, we followed asymptomatic patients until they reached a minimum weight of 7 kg or 4 months of age. We explained the surgical options (open or robotic) to the parents and proceeded with the surgery based on their decision. Based on our data, we propose an algorithm to determine the appropriate surgical timing for patients with asymptomatic CCs (Figure 4).

In our study, the AST, ALT, total bilirubin, direct bilirubin, and GGT levels in symptomatic patients were higher than those in asymptomatic patients. This result is similar to that of the study from the Netherlands (9). An advantage of our study, compared with previous studies, was a sufficient number of patients to allow better confirmation of significant results. However, our results should be interpreted carefully to avoid generalization to all patients. Previous studies have reported that grades III and IV liver fibrosis were significantly more common in the LSG of pediatric patients with CCs (10). However, Sugandhi et al. (27) reported that postoperatively, significant resolution of histological changes was observed in hepatocellular damage, parenchymal inflammation, cholestasis, and bile duct proliferation. In our study, which compared the ESG and LSG, the postoperative serum liver enzymes showed normalization and function recovery, with no significant difference. Furthermore, based on our study results regarding liver function recovery, it could be suggested that appropriate surgical intervention can prevent irreversible liver damage.

A limitation of our study was its retrospective single-institution design and inclusion of patients who transferred after prenatal diagnosis. It may be possible to have bias in patient selection and a higher rate of referrals after prenatal diagnosis. In addition, at antenatally diagnosis, the CDB size was not clearly recorded, and bias in the timing of each surgical decision may be present. Future a multicenter prospective study is needed to determine the optimal timing of surgery in patients with prenatal diagnoses of CCs.

## 5. Conclusion

The results of this study showed a longer hospital stay, longer duration of diet restriction, and higher rate of postoperative complications in patients in the ESG than in the LSG. However, liver function recovery was not different between the groups. Based on these results, asymptomatic patients should be closely monitored, and we recommend that definitive surgical intervention be postponed until 4 months of age or weight up to 7 kg.

## Data Availability

The original contributions presented in the study are included in the article/Supplementary Material, further inquiries can be directed to the corresponding author.
